# *Salmonella*-Based Vaccine: A Promising Strategy for Type 1 Diabetes

**DOI:** 10.3390/vaccines13040405

**Published:** 2025-04-14

**Authors:** Mahmoud Singer, Fouad Kandeel, Mohamed I. Husseiny

**Affiliations:** 1 Department of Radiological Sciences, School of Medicine, University of California Irvine, Irvine, CA 92697, USA; 2 Department of Translational Research and Cellular Therapeutics, Artur Riggs Diabetes & Metabolism Research Institute, Beckman Research Institute, City of Hope National Medical Center, Duarte, CA 91010, USA

**Keywords:** *salmonella*-based vaccine, oral vaccine for T1D, SPI2-T3SS of *Salmonella*, combination therapy, immunomodulator, tolerance

## Abstract

Type 1 diabetes (T1D) is a chronic autoimmune disease characterized by the progressive destruction of insulin-producing β-cells in the pancreas. Currently, no therapy exists to halt or cure T1D. Vaccination with diabetic autoantigens may offer protection against T1D development. Genetically modified, attenuated *Salmonella* utilizing the *Salmonella*-Pathogenicity Island 2 (SPI2)-encoded Type Three Secretion System (T3SS) can elicit robust immune responses, making it an attractive vaccine platform. Using SPI2-T3SS to deliver an autoantigen alongside immunomodulators and anti-CD3 antibodies induces antigen-specific regulatory T-cells. Our preclinical studies demonstrated the efficacy of a *Salmonella*-based vaccine in both preventing and reversing autoimmune diabetes in non-obese diabetic (NOD) mice while also exploring its genetic modifications, underlying mechanisms, and delivery strategies. This review evaluates the advantages of an oral T1D vaccine employing live, attenuated *Salmonella* for autoantigen delivery. We also discuss future directions for advancing this strategy in the treatment of other autoimmune diseases.

## 1. Introduction

Type 1 diabetes (T1D) results from dysregulated immune cell destruction of the insulin-producing β-cells of the pancreatic islets [1,2,3]. At present, treatment for T1D focuses on symptoms management, primarily through multiple daily insulin injections to control blood sugar levels. However, the growing incidence of T1D, its progressive complications, and the absence of curative or preventive strategies underscore the need for innovative therapies that can restore immune tolerance. At present, no safe, effective treatment exists to control the onset and progression of this lifelong debilitating disease [4]. Therapeutics that are antigen-specific for protection against autoimmunity may be applicable.

β-cell destruction in T1D is mediated principally by CD4^+^ and CD8^+^ T lymphocytes. Autoreactive cytotoxic effector CD8^+^ T lymphocytes are thought to be involved in mediating islet β cell destruction in T1D-like non-obese diabetic (NOD) mice and in humans [5]. Loss of regulation results in the appearance of autoantigen-specific antibodies and cytotoxic T cells (CTLs) that mediate the autoimmune killing of pancreatic insulin-producing β cells [6]. Evidence suggests that proinsulin (PI) is a main initiator of autoantigen sensitivity in NOD mice [7,8], and humans [9,10]. Epitope spreading then leads to new autoantigen sensitivity to glutamic acid decarboxylase (GAD65) [11], islet-specific glucose-6-phosphatase (IGRP) [12], and zinc transporter 8 (ZnT8) [13], eventually resulting in loss of metabolic control because of insulin insufficiency. Another contributing factor to this immune dysregulation is defective antigen-presenting cells (APCs), particularly dendritic cells (DCs), in the gut, which fail to support the differentiation of gut associated regulatory T cells (Tregs) [14] that modulate the response to self-antigens. Regulatory T cells are crucial for maintaining immune system balance under normal conditions, but their function is impaired in cancer and autoimmune diseases [15]. Modulating the pancreatic microenvironment to halt the autoimmune response is a goal in designing a protective vaccine that can prevent this destructive reaction. One approach utilized the SPI2-T3SS of live attenuated *Salmonella* to express and deliver autoantigens while also delivering plasmids that enabled host cells to produce tolerogenic cytokines in the gut, creating a microenvironment that prevented autoimmune diabetes.

## 2. Clinical Trials for Type 1 Diabetes Therapies: Challenges and Limitations

Ongoing clinical trials are exploring vaccine-based therapies for T1D in multifaceted approaches at modulating the immune system to preserve or restore pancreatic β-cell function.

The potential of the Bacillus Calmette-Guérin (BCG) vaccine, traditionally used for tuberculosis, as a T1D treatment is currently under investigation at Massachusetts General Hospital (MGH). A Phase I trial demonstrated that two BCG injections significantly lowered HbA1c in individuals with T1D, without causing hypoglycemia episodes. BCG vaccine induces an immune system reset and alters glucose metabolism, shifting energy production from low-sugar oxidative phosphorylation to high-sugar aerobic glycolysis [16]. Another proposed mechanism is that BCG enhances monocytes metabolism by improving defective glucose transport [17]. Despite its potential, the BCG vaccine has several limitations, including inconclusive clinical evidence, safety concerns in immunocompromised individuals, limiting long-term outcome data, and the need for further research to clarify its mechanisms of action and determine the optimal dosing strategy [18,19].

Proinsulin peptide-loaded, autologous tolerogenic DCs (PIpepTolDC) represent a promising therapeutic strategy against T1D. A Phase I clinical trial evaluated the safety and feasibility of intradermal administration of modified DC in T1D patients. The treatment was well-tolerated, with no serious side effects observed. Moreover, the intervention may modulate immune responses, as indicated by altered T cell reactions to proinsulin [20,21]. While PIpepTolDC hold promise, its use presents several drawbacks, including concerns over insufficient tolerance induction, immune response variability between patients, challenges in both manufacturing and administration, and the possibility for adverse immune reactions in certain individuals. These factors contribute to its limitations as a universally effective treatment for T1D [21,22,23,24].

A study was conducted to evaluate the potential of influenza vaccination in preserving β-cell function in children and adolescents with recent-onset T1D. It is hypothesized that the vaccine may alter the autoimmune response characteristic of T1D through immune modulation. The results have not yet been published [25].

IMCY-0098 is a synthetic peptide immunotherapy derived from human proinsulin, designed to prevent the progression of T1D by specifically eliminating pathogenic T cells. Unsupervised machine learning analysis, powered by artificial intelligence, revealed that patients with the HLA-DR4 haplotype had better clinical outcomes, including sustained C-peptide levels and reduced insulin requirements. The results from this trial are yet to be published [26]. IMCY-0098 requires additional preclinical mechanistic research to ensure proper evaluation in clinical trials.

Clinical trials are investigating the combination of immune suppression to control autoantigen specific CTLs, which result from defects in recessive tolerance, and the restoration of Treg function as a potential treatment for T1D. The aim is to preserve and restore the insulin-producing β-cell mass and function [27]. Anti-CD3 therapy targets the T cell receptor to preserve pancreatic β-cell function and delay disease progression [28,29]. The FDA recently approved the anti-CD3 antibody (Teplizumab) to delay the onset of T1D [30,31]. Teplizumab works by slowing the autoimmune destruction of β-cells, helping to prolong insulin production and reduce the need for exogenous insulin, which can improve glycemic control and lower the risk of long-term diabetes complications [32,33]. However, anti-CD3 therapy has drawbacks, including transient side effects such as cytokine release syndrome, flu-like symptoms, and an increased risk of infections due to immune modulation. Its effectiveness varies among patients, and it does not provide a permanent cure for T1D. Additionally, the high cost and need for careful patient selection limit its widespread use [28]. While this therapy has successfully reversed diabetes in rodents, its effects in humans are partial and temporary [34]. Patients receiving anti-CD3 antibodies show short-term improvements, such as prolonged C-peptide response and reduced insulin requirements, but these benefits diminish after two years [30,31].

Vaccination with diabetes-specific autoantigens is expected to increase antigen-specific Tregs and promote immune tolerance [15,35,36]. This approach has successfully reversed diabetes in animal models [37]. DNA vaccines expressing human proinsulin aim to modulate the autoimmune response in T1D by preserving pancreatic β-cell function. Clinical results indicate the preservation of C-peptide levels and a reduction in insulin-specific CD8+ T cells without significant adverse effects [38]. However, the benefits diminish after treatment cessation, highlighting the need for continuous administration or adjunctive therapies. While the current situation is promising, further research is needed to optimize dosing and assess long-term efficacy and safety in diverse populations [38,39,40,41].

As a result, experts are optimistic that antigen-specific immunotherapies, in combination with other treatments, could effectively help reverse autoimmunity while reducing side effects.

## 3. *Salmonella* as a Carrier for Vaccine Delivery

The *Salmonellae* genus includes two species: *Salmonella enterica* and *Salmonella bongori*. *S. enterica* is divided into seven subspecies (I, II, IIIa, IIIb, IV, VI, and VII). Subspecies I account for nearly 99% serovars associated with human disease, including S. *typhi*, which causes typhoid fever, and *S. enteritidis*, for a leading cause of gastroenteritis [42].

**Figure 1:**
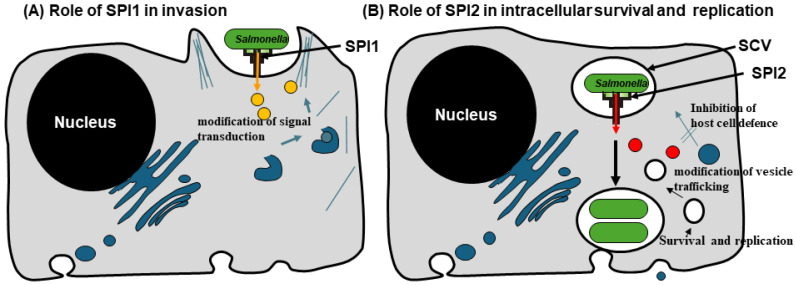
*Salmonella* pathogenicity islands encoding type three secretion system (SPIs-T3SS). (**A**) The SPI1 plays a pivotal role in *Salmonella* pathogenesis by mediating the invasion of non-phagocytic epithelial cells. It encodes a T3SS, which translocates effector proteins into host cells, promoting bacterial entry and infection. This strategy is utilized by extracellular bacteria to successfully gain entry into host cells and establish infection. (**B**) The SPI2 plays a crucial role in the intracellular survival and replication of *Salmonella* bacteria within host cells. SPI2 secretes effector proteins that alter host cell signaling, modulate *Salmonella* containing vacuole (SCV) membrane dynamics, and promote nutrient uptake, enabling *Salmonella* to successfully replicate within host cells. This figure was created using BioRender.com and assembled in Microsoft PowerPoint.

*Salmonella* interaction with host cells allows a safe delivery of a vaccine payload to the targeted epithelial cells in the gut through the hereafter mechanism. *Salmonella* invades host cells and move into membrane-bound vacuole termed *Salmonella*-containing vacuoles (SCV) [43]. The vacuoles maturate through extensive interactions with the endo lysosomal trafficking pathway. Afterwards, it interacts with host cells through the activity of bacterial effector proteins that are transported into host cells via type III secretion systems (T3SS) [44]. Invasion of epithelial cells and the initial steps of SCV biogenesis are linked to the T3SS encoded in the *Salmonella* pathogenicity island-1 (SPI-1 T3SS) (Figure 1A). Meanwhile, the *Salmonella* pathogenicity island-2 T3SS (SPI-2 T3SS) secretes effector proteins that are essential for SCV maturation, bacterial replication, and intracellular survival (Figure 1B) [45]. The features render *Salmonella* an appropriate choice for vaccine delivery.

### 3.1. SPI-1 T3SS: An Essential System for Vaccine Delivery

SPI-1 is expressed early in the infectious stage (Figure 1A) and is responsible for translocating effector proteins to manipulate key host–cell functions, such as signal transduction, cytoskeletal architecture, membrane trafficking and cytokine expression to the advantage of the pathogen [46,47]. Interestingly, a SPI-1 T3SS deficient strain (*hilA::tet*) was lower efficient than wild type strain in colonizing the intestine [48,49]. It was hypothesized that SPI-1-dependent colonization of the intestine is important during *Salmonella*–host interactions [49]. SopD is a protein of T3SS system that modulating the activity of Rab GTPases, which are crucial regulators of intracellular trafficking of *Salmonella typhimurium* and the progression of gastroenteritis [50].

### 3.2. SPI-2 of Salmonella: In Vivo–Inducible Promoters–A Double Edge Weapon

The function of SPI-2 is crucial for the second feature of *Salmonella* pathogenesis (Figure 1B), which is the ability to cause systemic infections and undergo proliferation within host organs. This remarkable attribute is linked to the potential of *Salmonella* to survive in phagocytic cells and to multiply within *Salmonella*-containing vacuoles (SCVs) [51]. The specific intracellular expression of heterologous antigens by *Salmonella* results in the stimulation of the immune responses [52,53]. Studies have shown that selecting an in vivo-activated (SPI2) promoter is needed in constructing efficient recombinant vaccines [54].

## 4. Innovative Use of *Salmonella* as a Vaccine Delivery Platform

*Salmonella* is an attractive organism for the generation of live attenuated vaccines [55]. The use of live attenuated *Salmonella* to translocate diabetogenic autoantigens to APCs via the SPI-2 T3SS is also a promising strategy for vaccine antigen delivery in T1D [56,57]. When given orally, *Salmonella* carrying a plasmid for autoantigen-expression is taken up by APCs in the gut, such as DCs. Inside these cells, the bacteria form SCV, where they survive, replicate, and deliver the recombinant antigen into the host cytosol, thereby bypassing intestinal degradation [56]. The T3SS of *Salmonella* consists of more than 20 proteins and are among the most complex protein secretion systems known in bacteria [58]. These are complex molecular machines that mediate the translocation of effector proteins from the *Salmonella* cytoplasm into the cytoplasm of infected host cells, acting like molecular syringe needles [59,60,61]. The SP12-T3SS is specifically induced by intracellular *Salmonella* and mediates the translocation of numerous effector proteins across the phagosomal membrane enclosing the pathogen (Figure 1B) [56]. Live attenuated *Salmonella* utilizing SPI2-T3SS offers a promising approach for developing recombinant vaccines against infectious diseases and cancer [62,63]. In T1D, the same approach was used to express the anti-inflammatory genes related to immune suppression [64,65,66,67].

### 4.1. Induction of Oral Ttolerance via Salmonella-Based Vaccine

Oral tolerance is the dynamic process by which the immune system fails to respond to an orally administered antigen. For inducing tolerance, the oral delivery of antigens has consistently shown its effectiveness over time [68]. The GALT encounters antigens arising from the intestinal flora and dietary intake and still prevents inflammatory responses by its tendency toward tolerance. This tendency has been exploited in the development of promising treatments for autoimmune diseases [41].

The mucosal immune system has developed several mechanisms to tolerate self-antigens and environmental antigens present in air, the microbiome, and food [41]. These strategies include anergy, activation-induced apoptosis, and the induction of regulatory T cells [40,57]. Induction of regulatory cells following mucosal antigens delivery was reported in animal models for over 40 years and has received attention for its potential role in regulating immune-mediated diseases [41,69,70].

The development of a genetically modified live attenuated *Salmonella* could offer a way forward towards immune balance in T1D. The use of attenuated *Salmonella* expressing proinsulin, delivering plasmids for TGF-β and IL10 expression, combined with a low-dose of anti-CD3 antibodies, was effective at treating T1D in NOD mice through immune modulation, promoting tolerance in tissue-resident T cells and APCs [64,65,66,67]. The oral *Salmonella*-based vaccine induced immune tolerance by increasing regulatory cytokines IL-10, IL-2, IL-13, and MCP-1, while reducing inflammatory cytokines IFN-γ, GM-CSF, TNF-α, IL-7, and IL-12 [71,72]. Additionally, the oral *Lactococcus*-based vaccine secreted IL-10, reducing immune cell infiltration into the islets and preventing or slowing diabetes progression [73,74,75].

### 4.2. Mechanism of Oral Salmonella-Based Vaccine

**Figure 2:**
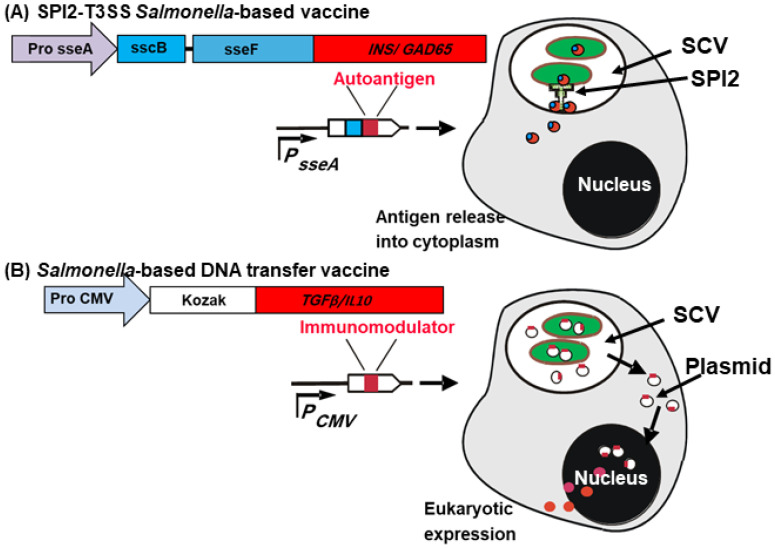
Rational design of *Salmonella* -based vaccines for autoimmune diabetes. (**A**) The T3SS-facilitates cytosolic delivery of aSPI2 effector (SseF) fused to an autoantigen (proinsulin or GAD65), under the control of an in vivo-inducible promoter (*Pro sseA*), enables targeted antigen presentation. (**B**) In the DNA transfer approach, *Salmonella* delivers plasmids encoding antigens under control of a eukaryotic promoter. Host cells then express and secrete cytokines (TGFβ and IL10) under the control of the cytomegalovirus promoters (*Pro CMV)*. Since *Salmonella* cannot escape the *Salmonella*-containing vesicle (SCV), the plasmid DNA is released into the phagosome and may enter the nucleus for gene expression. This figure was created using BioRender.com and assembled in Microsoft PowerPoint.

Our team developed an oral *Salmonella*-based delivery system in which a diabetic autoantigen (mouse PI or human GAD65) was fused with the *Salmonella* SPI2 effector (sseF), which was expressed under the SPI2 promoter and translocated into the cytosol of infected cells (Figure 2) [76,77]. This system was combined with *Salmonella*-delivered plasmids expressing the immunomodulators TGFβ and IL10, which were secreted under the control of a CMV promoter by host cells (Figure 2) [56]. The *Salmonella*-based oral vaccine, which expresses autoantigens and is combined with tolerogenic cytokines and a sub-therapeutic dose of anti-CD3 mAb, successfully prevented and reversed autoimmune diabetes in NOD mice [64,65,66,71].

Combination therapy induced antigen-specific tolerance in a diabetic NOD mice model [57,78]. The loss of immune tolerance underlying T1D was addressed using autoantigen-specific therapies, which had fewer adverse-effects than non-specific immunosuppression [79].

Oral bacteria-based approaches have been explored as vectors for antigen-specific immunotherapies for diabetes. The co-administration of anti-CD3 induced antigen-specific tolerance and reversed early onset diabetes [65,71,78]. By adopting combination therapy in the *Salmonella* vaccine model, it simultaneously influenced different immune pathways. For example, it increased the frequency of CD4^+^CD25^+^Foxp3^+^ Tregs in the spleen and pancreatic lymph nodes (PLNs) of NOD mice, along with a concomitant increase in the frequency and function of CD4^+^CD49^+^LAG3^+^ Tr1 cells [64,65,71,72].

**Figure 3:**
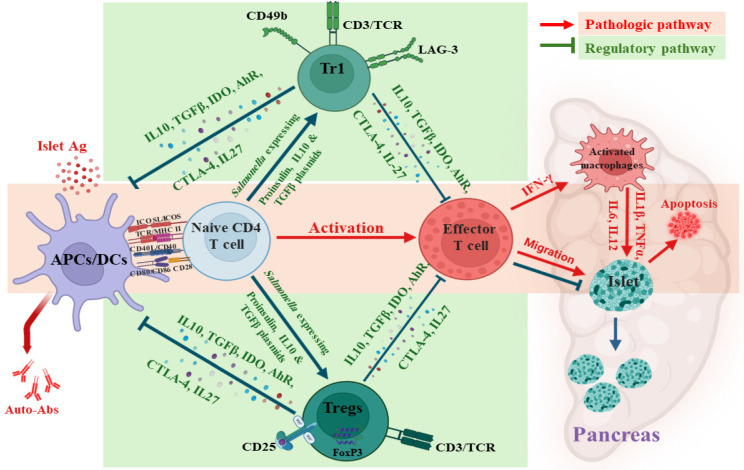
The pathological mechanisms and therapeutic approaches in T1D. In the pathological mechanisms of T1D (in red), APCs present islet-specific antigens to naïve CD4^+^ T cells, leading to the activation of effector CD4^+^ and CD8^+^ T cells. These activated T cells migrate to the pancreatic islets, where they contribute to the destruction of insulin-secreting β-cells. Effector T cells release pro-inflammatory cytokines, such as IFN-γ, which promote macrophage activation, resulting in islet damage and β-cell apoptosis, leading to the development of T1D. In the regulatory approaches (in green), *Salmonella* expressing proinsulin, combined with plasmids encoding IL10 and TGFβ, enhances Tregs and Tr1 cells. These cells release anti-inflammatory cytokines like IL10, TGFβ, and IDO, which counteract effector T cell activation and protect pancreatic islets. This figure was created using BioRender.com and assembled in Microsoft PowerPoint.

The efficacy of a *Salmonella* vaccine to prevent and reverse T1D was assessed in NOD mice [71]. By orally administering the vaccine combined with anti-CD3 antibodies to mice with acute and progressive onset of diabetes, we found that 63% of acute and 78% of progressive diabetic mice exhibited lower blood glucose levels, along with a remarkable increase in insulin positive cells after therapy. Surprisingly, the vaccine induced tolerance in all vaccinated mice, regardless of the timing of the disease onset. Older vaccinated (progressive) mice had higher *IL-13* and lower *IL-1α* and *CXCL5* levels than younger (acute) mice. In addition, vaccinated mice with progressive disease exhibited less islet inflammation and more β-cells than those with acute disease onset. These findings indicate that the vaccine can reverse diabetes post-onset and that some cases, early-stage disease preserves residual β-cell function [71]. These findings highlight the importance of therapy timing, as it is directly affected by the progression of the disease. For example, individuals diagnosed at an older age exhibited a slower decline in C-peptide levels, reflecting preserved β-cell function [80]. Direct measurement of β-cell death, such as detecting circulating levels of unmethylated insulin DNA, could provide greater insight into the relationship between intervention strategies and clinical outcomes [81,82]. Investigation of the immune mechanism revealed that the vaccine increased the frequency of antigen-specific suppressor CD4+ CD25+ Foxp3+ Treg cells and CD4+ CD49b+ LAG3+ type 1 regulatory (Tr1) cells in lymphoid orangs [64,65,71,72]. Vaccinated mice exhibited antigen-specific suppressor Tregs, Tr1 cells, and tol-DCs in their lymphoid organs, all of which suppressed responder T cell proliferation (Figure 3). Alterations in inhibitory gene expression within Tregs and tol-DCs may contribute to their immunosuppressive activity. Immune suppression was not limited to regulatory T cells but also extended to antigen-presenting cells. The vaccine increased the presence of myeloid DCs in lymphoid organs.

These myeloid DCs, characterized by lower MHCII and CD80 expressions, contributed to an immunotolerant microenvironment in lymphoid organs near the pancreas. From a different analytical perspective, the *Salmonella* vaccine led to increased expression of the immune checkpoint molecules CTLA-4 and PDL-1. Furthermore, increase expression of aryl hydrocarbon receptor (AhR), which inhibits pro-inflammatory cytokine production and dampens autoimmune activity, was observed [83]. In DCs, the vaccine upregulated indoleamine 2,3-dioxygenase (IDO), an immune checkpoint produced by activated macrophages and Tregs to induce immune tolerance [84,85,86]. Although increased expression of CTLA-4, PD-L1, AhR, and IDO may help reduce diabetes-related inflammation, excessive expression of these molecules could suppress T cell function and induce T cell apoptosis (Figure 3) [87]. The intriguing effect of the vaccine to increase DC-SIGN modulated DCs activity likely influenced its interactions with immune checkpoints and antigen presentation. This, in turn, fostered immune tolerance, especially toward self-antigens [88,89]. Moreover, the *Salmonella*-based vaccine stimulated increased production of IL-27 from DCs and macrophages [90]. IL-27 may help regulate the immune response to prevent or mitigate the autoimmune attack on β-cells [90]. Additionally, IL27 enhanced the development and function of Tregs in autoimmune T1D [91]. The vaccine appeared to induce localized immune suppression to regulate the autoimmune response without compromising the overall immune defenses.

## 5. Advantages of *Salmonella*-Based Vaccines

Live attenuated vaccines have several advantages over other types of vaccines, including ease of administration, the ability to carry heterologous antigens, and the capacity to induce mucosal, cellular, and humoral immune responses [92]. *Salmonella* is an interesting organism able to act as a carrier for the delivery of recombinant antigens and immunomodulators [55]. Vaccines require a careful and detailed immunological analysis as related to design and delivery [93]. *Salmonella* is an ideal vector for tolerogenic vaccine development for several reasons including the natural history of this genus and what it means for effective delivery of antigens and other effectors and the ease to produce safe and effective oral vaccines. Fusion of heterologous antigens to specific SPI-2 proteins causes them to be passed directly into the cytosol of the APCs where the antigens are processed and presented to immune cells within the gut mucosa [94,95], bypassing expression of antigen in the intestinal lumen avoids degradation, loss of antigen, and unwanted immune responses. A second way in which *Salmonella* can influence the host APC is by carrying mammalian expression plasmids. This feature has been used to develop DNA vaccines [96,97] but it can also be used to carry tolerogenic cytokines that are directly expressed by the host cell creating a local immune privileged microenvironment. Oral administration of *Salmonella*-based vaccines is convenient, requiring only 2 doses [57]. In contrast, a *Lactococcus*-based vaccine must be administered 5 times per week for 6 weeks [78,98]. Animal studies support the *Salmonella* vaccine strategy [99]. Additionally, an FDA-approved oral *Salmonella*-based vaccine (Vivotif, PaxVax, Redwood City, CA) that is currently manufactured and sold in the United States. This vaccine has been used worldwide for decades, with over 150 million doses administered and no major adverse events reported [100].

## 6. Limitation of *Salmonella*-Based Vaccine, Risks, and Mitigation Strategies

Although *Salmonella*-based vaccines provide a promising platform for developing vaccines against various infectious diseases, they have several limitations that must be overcome for broader application. One major concern is the genetic instability of attenuated *Salmonella* strains, which can potentially revert to virulent forms through mutations or recombination events [101]. The attenuated *Salmonella* strain may lose the expression cassette (plasmid) during infection in the absence of a selective antibiotic. To circumvent this, a method for stable and efficient expression of heterologous antigens by integrating the antigen into the *Salmonella* chromosome was established [94,95]. This approach serves as an alternative strategy to enhance vaccine stability and therapeutic efficacy.

Live attenuated *Salmonella* (*ΔhtrA*/*ΔpurD*) was used as vaccine carrier; however, it retains the outer membrane lipopolysaccharide (LPS), which may induce endotoxic septic shock [56,57,64,76]. To address this, LPS-deficient, FDA-approved *Salmonella typhi* Ty21a [102], O-antigen-deficient *S. typhimurium ΔrfaD* [103], or lipid A-deficient *Salmonella ΔmsbB* [103,104] strains could be used and warrant further testing.

Autoantibodies are present in the most individuals with T1D and those at high risk of developing the disease [105]. Insulin, GAD65, and IA-2 antibodies are detected in 95% of pre-diabetic and newly diagnosed T1D patients [106]. At least two of these autoantibodies are positive in 80% of individuals with T1D. In contrast, such autoantibodies are rarely, if ever, found in non-diabetic individuals [107]. The presence of autoantibodies is a strong predictor of T1D development [108], with the risk increasing as more autoantibodies appear [106]. This is critical for vaccine development, as targeting a single islet autoantigen is unlikely to be sufficient for preventing or treating T1D. To overcome this, a *Salmonella* vaccine could incorporate multiple autoantigens or their epitopes to target a repertoire of T1D-associated autoantigens.

Certain antigen epitopes exhibit a low affinity for binding to MHC class II molecules on APCs [109], contributing to the loss of self-tolerance in T1D [110]. This weak binding impairs the elimination of autoreactive T cells in the thymus and fails to sufficiently increase Treg cell expansion and activation [111]. Modifying the InsB9-23 peptide by introducing a single amino acid change (R22E) [79] or two amino acid changes (E21G-R22E) [112] significantly enhanced MHC II binding. Incorporating these insulin-derived mimotope peptides into a *Salmonella* vaccine is expected to further improve efficacy.

NOD mice are widely used to study the mechanisms underlying autoimmune diabetes. However, the disease presents in a less heterogeneous manner than in humans, which may be misleading and impact the development of immune-based therapies [113]. Therefore, evaluating the vaccine in context of a human immune response is crucial. A humanized mouse model of T1D was reported [112,114,115] but, at this time, is not available. Such mice carried human immune cells [116,117] and an effort to develop version of this model and share it widely would seem reasonable.

## 7. The Future of *Salmonella*-Based Vaccines: Challenges and Opportunities

The development of *Salmonella*-based vaccines for T1D is gaining traction as researchers explore novel immunotherapeutic strategies to modulate autoimmunity before clinical onset [56]. The use of *Salmonella* as an oral delivery system offers a significant advantage by stimulating mucosal immunity, an essential factor in systemic immune modulation for T1D prevention [72].

Research has focused on optimizing *Salmonella* vectors for improved safety and efficacy in clinical trials. Genetic modifications aim to reduce pathogenicity while retaining antigen presentation [118], including deleting virulence genes, controlling antigen expression, and enhancing colonization of gut-associated lymphoid tissue without causing systemic infection. These refinements help maintain immunogenicity and minimize risks, which is critical for regulatory approval process before human trials [65,119].

Before starting clinical trials, scalability and manufacturing consistency are critical factors for attenuated *Salmonella* vaccines. Production must meet Good Manufacturing Practices (GMP) for stability, purity, and reproducibility [120]. The oral delivery route requires strategies to improve bacterial survival in the gastrointestinal tract while maintaining immunogenicity. Advances in encapsulation technologies, like biofilm coating or polymer microencapsulation, are being investigated to enhance vaccine viability and efficacy [121,122]. The City of Hope has GMP core facilities to produce clinical-grade products, including bacterial vectors such as the *Salmonella* and antibodies for T cell depletion.

As *Salmonella*-based vaccines move closer to clinical trials, regulatory and ethical considerations will be crucial. Preclinical toxicology studies must confirm safety, addressing concerns about bacterial persistence and unintended immune effects [71]. Collaboration between researchers, industry, and regulatory agencies will be essential to advancing these vaccines. If successful, *Salmonella*-based vaccines could revolutionize T1D immunotherapy, providing a therapeutic strategy for other autoimmune diseases. In fact, the FDA-approved oral *Salmonella* vaccine is already being used worldwide, including in the United States, could help ease the regulatory process for clinical translation.

## 8. Conclusions

A *Salmonella*-based vaccine for T1D demonstrated efficacy in stopping, preventing, and reversing the destruction of pancreatic β-islet cells. Immunological studies showed that tolerance was primarily mediated by regulatory T cells and antigen-presenting cells. Microbiomic and metabolomic analyses showed favorable changes in response to the vaccine. However, long-term safety assessments and further cytokine optimizations are needed to enhance outcomes. The *Salmonella*-based T1D vaccine could be combined with other novel T1D therapies, such as stem cells, exosomes, and or locoregional immunomodulatory treatments, to promote pancreatic cell regeneration. Such an approach integrates both immunomodulatory and regenerative therapies. A similar strategy could potentially be applied to other autoimmune diseases.
